# The Elusive Baseline of Marine Disease: Are Diseases in Ocean Ecosystems Increasing?

**DOI:** 10.1371/journal.pbio.0020120

**Published:** 2004-04-13

**Authors:** Jessica R Ward, Kevin D Lafferty

**Affiliations:** **1**Department of Ecology and Evolutionary Biology, Cornell UniversityIthaca, New YorkUnited States of America; **2**United States Geological Survey Western Ecological Research Center, Marine Science InstituteUniversity of California, Santa Barbara, Santa Barbara, CaliforniaUnited States of America

## Abstract

Disease outbreaks alter the structure and function of marine ecosystems, directly affecting vertebrates (mammals, turtles, fish), invertebrates (corals, crustaceans, echinoderms), and plants (seagrasses). Previous studies suggest a recent increase in marine disease. However, lack of baseline data in most communities prevents a direct test of this hypothesis. We developed a proxy to evaluate a prediction of the increasing disease hypothesis: the proportion of scientific publications reporting disease increased in recent decades. This represents, to our knowledge, the first quantitative use of normalized trends in the literature to investigate an ecological hypothesis. We searched a literature database for reports of parasites and disease (hereafter “disease”) in nine marine taxonomic groups from 1970 to 2001. Reports, normalized for research effort, increased in turtles, corals, mammals, urchins, and molluscs. No significant trends were detected for seagrasses, decapods, or sharks/rays (though disease occurred in these groups). Counter to the prediction, disease reports decreased in fishes. Formulating effective resource management policy requires understanding the basis and timing of marine disease events. Why disease outbreaks increased in some groups but not in others should be a priority for future investigation. The increase in several groups lends urgency to understanding disease dynamics, particularly since few viable options currently exist to mitigate disease in the oceans.

## Abstract 2

Reports of disease in the scientific literature, normalized to overall publication rates, detect important — and some unexpected — trends of disease in major groups of marine plants, invertebrates, and vertebrates

## Introduction

Marine organisms serve as hosts for a diversity of parasites and pathogens. Mortalities affect not only the host population, but can cascade through ecosystems. Loss of biologically engineered habitats such as seagrass beds (Lewis 1933; Taylor 1933) and cascading trophic effects due to removal of consumers (Lessios 1988) can alter community structure.

Understanding marine disease and the timing of outbreaks is increasingly important given escalating anthropogenic stressors affecting marine ecosystems. Humans directly affect community structure (e.g., overfishing [Jackson et al. 2001; Myers and Worm 2003]) and facilitate introduction of terrestrial pathogens to marine organisms (e.g., canine distemper virus in Antarctic seals [Bengtson and Boveng 1991]). Human-mediated climate change may also affect disease prevalence. A recent review predicts disease in both terrestrial and marine ecosystems could increase with future climate warming (Harvell et al. 2002).

Previous literature reviews suggesting a higher rate of disease outbreaks in the last three decades (Epstein et al. 1998; Harvell et al. 1999), coupled with predictions of future increases due to climate change (Harvell et al. 2002), lend new urgency to understanding causes of marine disease outbreaks. Evidence suggests the increase is real (Harvell et al. 1999), yet lack of baseline data for most marine communities precludes a direct test of the hypothesis.

We developed a proxy method to test a prediction of the increasing disease hypothesis: that reports of disease in the scientific literature, normalized to overall publication rates, increased since 1970. We searched an online literature database (ISI Web of Science) and quantified reports of disease in natural populations of marine organisms from 1970 to 2001. Nine marine taxonomic groups were searched: turtles, corals, mammals, urchins, molluscs, seagrasses, deca-pods, sharks/rays, and fishes.

Previous analyses of ecological literature specifically assessed trends among scientists such as taxonomic bias (Clark and May 2002) and taxonomic chauvinism (Bonnet et al. 2002) in research. Our proxy method is to our knowledge the first quantitative use of normalized trends in the literature to investigate an ecological hypothesis. In the absence of baseline data, the literature proxy method detects important trends of disease in major groups of marine plants, invertebrates, and vertebrates.

## Results

The largest confounding factor when using literature searches to correlate disease events with time is temporal change in the total number of publications (related to disease or not) on the taxonomic group. To control for changes in total publication, data were normalized using a yearly proportion of disease reports from natural populations relative to total literature inputs for each taxonomic group.

**Figure 1:**
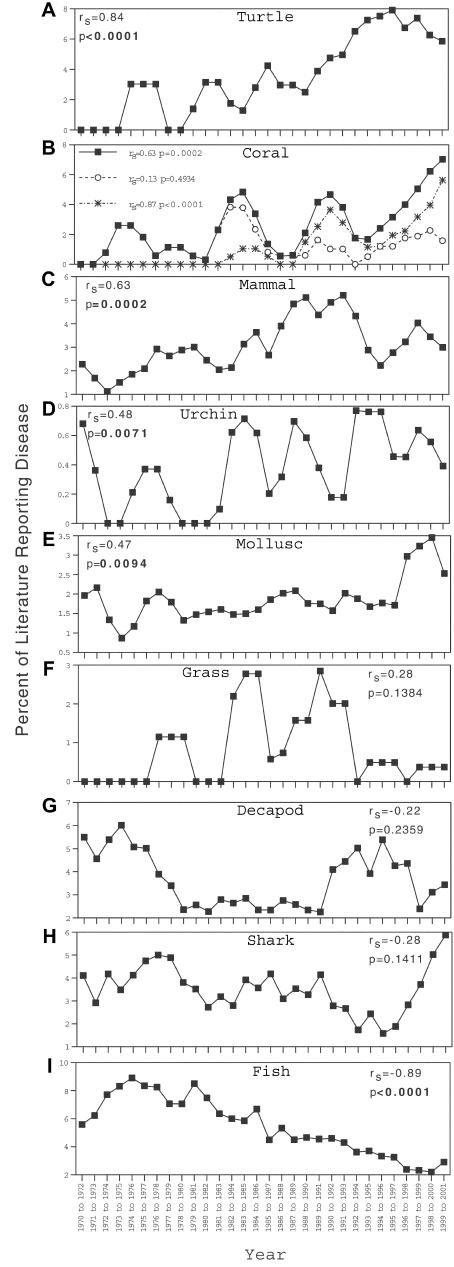
Percent of Literature Reporting Disease over Time in Each Taxonomic Group *r_s_* is Spearman's ρ. α is controlled for multiple comparisons with Holm's sequential Bonferroni adjustments. (A) Turtle. (B) Coral bleaching and disease (closed square); coral disease including infectious bleaching (open circle); coral bleaching (asterisk). (C) Mammal. (D) Urchin. (E) Mollusc. (F) Seagrass. (G) Decapod. (H) Shark/ray. (I) Fish.

Total disease reports, not normalized to literature inputs, increased in all groups (Table 1). However, normalized results varied with taxonomic group. Normalized disease reports increased in turtles, corals, mammals, urchins, and molluscs. No significant trends were detected for seagrasses, decapods, and sharks/rays (though disease occurred in these groups). Counter to the hypothesis, disease reports decreased in fishes (Figure 1).

**Table 1:**
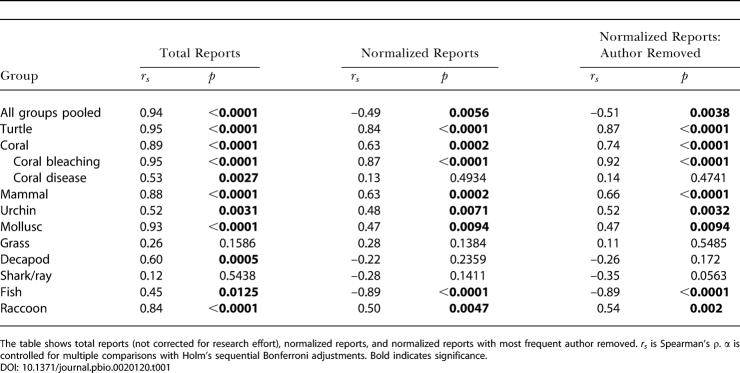
Spearman's Rank Correlation Analysis

The table shows total reports (not corrected for research effort), normalized reports, and normalized reports with most frequent author removed. *r_s_* is Spearman's ρ. α is controlled for multiple comparisons with Holm's sequential Bonferroni adjustments. Bold indicates significance

The relevance of our approach hinges on the assumption that an actual change in disease over time is accompanied by a corresponding change in publication frequency by scientists. We evaluated this assumption by testing the protocols with a case in which the baseline was known (raccoon rabies [Rupprecht and Smith 1994]). Normalized reports of raccoon rabies increased since 1970 (see Table 1) just as the disease increased from an index case in Virginia in 1977 to an epizootic affecting eight mid-Atlantic states and the District of Columbia by 1992 (Rupprecht and Smith 1994). Despite improvements in search protocols, use of a literature proxy is limited by the inability to distinguish between an event that did not occur and an event that was not reported.

We tested whether particular authors contributed disproportionate primary literature inputs that could bias results. Papers by the most prolific author in each taxonomic group were removed to determine whether there was an “author effect,” and none was observed in any taxonomic group (see Table 1). Multiple reports of a single disease event could also bias the data. Multiple reports were removed from the turtle, coral, urchin, mammal, shark/ray, and seagrass literature. Removal of the reports did not alter the significance of the results; thus, multiple reports in the mollusc, decapod, and fish literature were not removed, owing to the large volume of literature in these groups.

## Discussion

We address an ecological hypothesis, that disease of marine organisms increased since 1970, using a quantitative literature proxy method. Although total reports of marine disease increased over time (Epstein et al. 1998; see Table 1), a parallel increase in publication rates confounds interpretation of this pattern. Our approach normalizes data to overall publication within each group to circumvent this problem.

While an increase in disease reports was detected in many taxa, our finding that disease did not increase in all taxa has two important implications. First, the increases were not exclusively the result of increased study of disease by marine biologists. Second, factors such as global change may have complex effects on disease. Although some aspects of global change, such as warming and pollution, are predicted to make hosts more susceptible to infection (Scott 1988; Holmes 1996), some stressors may impact parasites more than their hosts (Lafferty 1997). Signs of infection with coldwater disease in salmonids, for example, occur between 4°C and 10°C and disappear as water temperature increases (Holt et al. 1989). In addition, stressors that depress host population density may reduce density-dependent transmission of host-specific infectious disease by reducing contact rates between infected and uninfected individuals (Lafferty and Holt 2003).

New or increasing stressors, such as global warming, could increase disease if stressed hosts are more susceptible to infection. Elevated sea surface temperature due to El Niño events is a common explanation for coral bleaching (Williams and Bunkley-Williams 1990; Hoegh-Guldberg 1999) and may increase coral susceptibility to disease (Harvell et al. 2001). Increases in turtle and mollusc disease also appear temperature-related. Green turtle fibropapilloma tumors are hypothesized to grow rapidly in summer and reach a debilitating size by winter, when cold water temperatures further stress turtles, resulting in winter strandings (Herbst 1994). The geographic range of the oyster parasite Perkinsus marinus extended 500 km north owing to an increase in average winter low temperatures (Ford 1996). Pollution is another ubiquitous and increasing stressor. Bioaccumulation of lipophillic toxins in marine mammals affects the immune system and increases susceptibility to disease (Lafferty and Gerber 2002).

Disease could also increase if transmission increases with host density. Some sea urchins experienced increased populations due to overfishing of their predators, and these high-density populations are associated with bacterial disease (Lafferty and Kushner 2000). Regulations such as the United States Marine Mammal Protection Act of 1972 fully protect pinniped populations, and several species have increased in abundance to levels where transmission efficiency would be expected to increase.

The decline in infectious diseases of wild fishes over time corresponds to documented reductions in fish populations through intense fishing (Jackson et al. 2001; Myers and Worm 2003). Fisheries that reduce the abundance of a fished species should also reduce infectious disease transmission (Dobson and May 1987). This has been documented in experiments (Amundsen and Kristoffersen 1990) and in observations of parasite declines associated with overfishing (Sanders and Lester 1981).

Grouping diseases within taxa could obscure important patterns. For example, the trend for increasing coral disease was driven by coral bleaching (*r_s_* = 0.87, *p* < 0.0001), while infectious coral diseases, including infectious bleaching, did not increase over time (*r_s_* = 0.13, *p* = 0.4934; see Figure 1B). The infectious bleaching literature includes several papers since 1996. To ensure the lack of a significant coral disease trend was not due to multiple papers published on this topic at the end of the time range surveyed, an additional analysis was conducted with all infectious bleaching papers excluded; *r_s_* and *p* values did not change (Table 2).

**Table 2:**
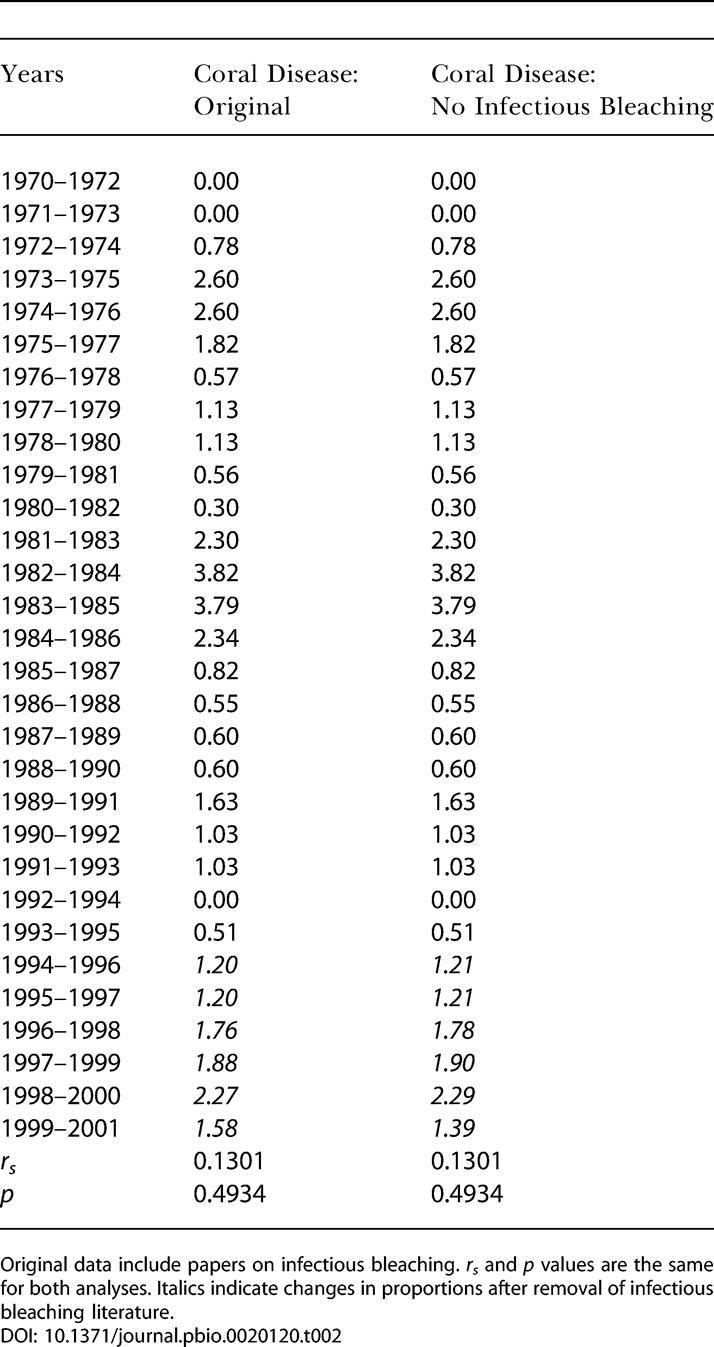
Normalized Coral Disease Reports

Original data include papers on infectious bleaching. *r_s_* and *p* values are the same for both analyses. Italics indicate changes in proportions after removal of infectious bleaching literature

While we did not detect an increase in normalized coral disease reports over time, impacts of disease can be high. The recent shift of dominant corals (*Acropora* to *Agaricia*) on reefs due to white band disease was unprecedented in the last 3,000 y (Aronson et al. 2002). Future research should take a finer-scale look at disease, particularly disease impacts, within each taxonomic group. Further investigation is also warranted to determine why some groups showed no temporal pattern in disease reports.

We examined temporal trends in disease reports since 1970 to identify groups experiencing increased outbreaks. The strong pattern of increased reports in groups such as turtles, mammals, and urchins reflects perceived changes noted by scientists (Harvell et al. 1999). Trends in other groups, such as seagrasses and fishes, suggest that an increase in disease did not occur across all taxa. Although this proxy approach does not directly test hypotheses of temporal changes in disease, a strong signal likely reflects an underlying pattern in nature. In the absence of baseline data, this is a useful approach for detecting quantitative trends in disease occurrence. Understanding disease dynamics, including trends in disease occurrence, is fundamental to conserve ecosystems faced with rising anthropogenic stresses.

## Materials and Methods

### 

We searched the Science Citation Index Expanded (5,900 journals, ISI Web of Science versions 1.1 and 1.2) for papers published from 1970 to 2001 with titles containing specific host taxonomic strings alone and in combination with a disease string (Table 3). We excluded articles clearly about disease in nonnatural settings, such as hatcheries, aquaculture, and mariculture, or about experimental or laboratory infections. Searches for corals were performed twice to quantify reports of bleaching separately from infectious bleaching (e.g., Vibrio shiloi [Israely et al. 2001]) and disease. Only titles were searched, as online abstracts are not available for many articles prior to 1990. Searching the complete citation would bias results after 1990 because more text of each publication would be searched.

**Table 3:**
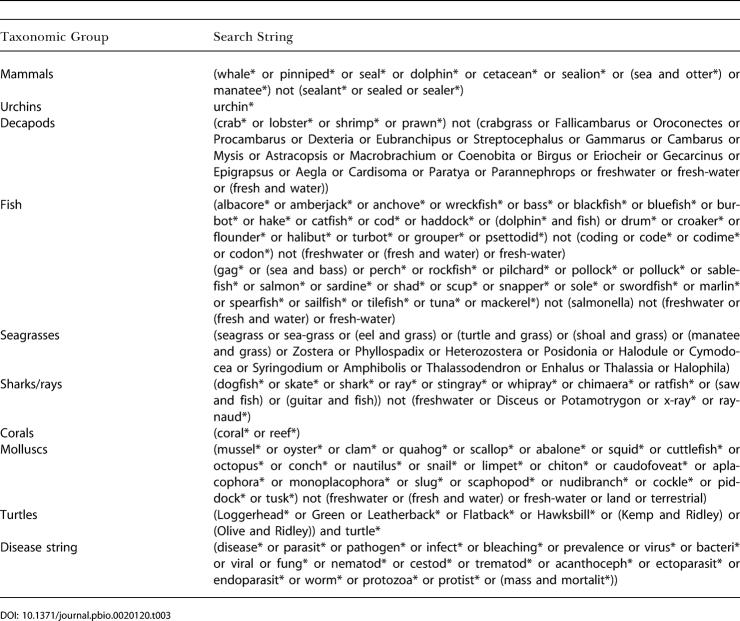
Taxonomic Groups and Search Strings

Abstracts (or entire manuscripts, when necessary) were obtained for articles within the turtle, coral, urchin, mammal, shark/ray, and seagrass literature that appeared to report the same disease event (e.g., multiple reports of the Caribbean *Diadema* urchin mortality). If more than one paper reported an event, only the earliest published report was included in the analysis. Because significance of results was not altered, multiple reports of disease were not removed from mollusc, decapod, and fish literature owing to the large number of publications returned for each group.

Often, returned titles contained part of the search string, but were not relevant (e.g. “crab nebula” when searching “crab”). Modifications to search strings excluded most irrelevant articles, and titles were read to determine relevance. If more than 50 titles were returned, titles were randomly sorted and the greater of 20% (maximum of 200) or 50 returned titles were read. Total relevant articles were calculated as the proportion of relevant articles read times the total number returned.

Protocols were tested using raccoon rabies, a disease for which baseline data are available (Rupprecht and Smith 1994). Potential biases were considered and tested. Extensive descriptive or taxonomic work early in the study of a group could bias results against a large number of disease reports. If such a bias existed, one would expect both a large number of disease reports and a large number of nondisease publications in the beginning of the literature survey period. Neither prediction is true—the number of both disease reports and nondisease publications either remains relatively constant or increases through time in all groups.

Frequent publishing by one author could bias results. Papers by the most published author in a taxonomic group were removed from the analysis to determine their effect. Papers on a particular “hot” topic could also bias results, particularly if that topic is disease and inflates normalized disease reports late in the survey period. For example, a recent mortality event could increase scientists' awareness of disease, resulting in increased publishing without a concomitant increase in the phenomenon. This likely does not affect our results because (a) disease is not the only “hot” topic experiencing increased publication rates and (b) while multiple papers on disease may be published, not all are reports of disease in natural populations.

A 3-y running mean was used to reveal trends obscured by clustered reporting (e.g., a symposium volume on a topic) and time lags between observation and publication (approximately 3 y, determined by comparing event and publication dates). Data were analyzed with Spearman's rank correlation (JMP version 5.0) with α controlled for multiple comparisons using Holm's sequential Bonferroni adjustments.
